# Connecting Network Properties of Rapidly Disseminating Epizoonotics

**DOI:** 10.1371/journal.pone.0039778

**Published:** 2012-06-25

**Authors:** Ariel L. Rivas, Folorunso O. Fasina, Almira L. Hoogesteyn, Steven N. Konah, José L. Febles, Douglas J. Perkins, James M. Hyman, Jeanne M. Fair, James B. Hittner, Steven D. Smith

**Affiliations:** 1 Center for Global Health, Health Sciences Center, University of New Mexico, Albuquerque, New Mexico, United States of America; 2 College of Veterinary Medicine, North Carolina State University, Raleigh, North Carolina, United States of America; 3 Faculty of Veterinary Science, University of Pretoria, South Africa, and Faculty of Veterinary Medicine, Utrecht University, Yalenaan, The Netherlands; 4 CINVESTAV, Mérida, Yucatán, México; 5 School of Science and Engineering, Tulane University, New Orleans, Louisiana, United States of America; 6 Los Alamos National Laboratory, Biosecurity & Public Health, Los Alamos, New Mexico, United States of America; 7 Department of Psychology, College of Charleston, Charleston, South Carolina, United States of America; 8 College of Agriculture and Life Sciences, Cornell University, Ithaca, New York, United States of America; Northeastern University, United States of America

## Abstract

**Background:**

To effectively control the geographical dissemination of infectious diseases, their properties need to be determined. To test that rapid microbial dispersal requires not only susceptible hosts but also a pre-existing, connecting network, we explored constructs meant to reveal the network properties associated with disease spread, which included the road structure.

**Methods:**

Using geo-temporal data collected from epizoonotics in which all hosts were susceptible (mammals infected by Foot-and-mouth disease virus, Uruguay, 2001; birds infected by Avian Influenza virus H5N1, Nigeria, 2006), two models were compared: 1) ‘connectivity’, a model that integrated bio-physical concepts (the agent’s transmission cycle, road topology) into indicators designed to measure networks (‘nodes’ or infected sites with short- and long-range links), and 2) ‘contacts’, which focused on infected individuals but did not assess connectivity.

**Results:**

The connectivity model showed five network properties: 1) spatial aggregation of cases (disease clusters), 2) links among similar ‘nodes’ (assortativity), 3) simultaneous activation of similar nodes (synchronicity), 4) disease flows moving from highly to poorly connected nodes (directionality), and 5) a few nodes accounting for most cases (a “20∶80″ pattern). In both epizoonotics, 1) not all primary cases were connected but at least one primary case was connected, 2) highly connected, small areas (nodes) accounted for most cases, 3) several classes of nodes were distinguished, and 4) the contact model, which assumed all primary cases were identical, captured half the number of cases identified by the connectivity model. When assessed together, the synchronicity and directionality properties explained when and where an infectious disease spreads.

**Conclusions:**

Geo-temporal constructs of Network Theory’s nodes and links were retrospectively validated in rapidly disseminating infectious diseases. They distinguished classes of cases, nodes, and networks, generating information usable to revise theory and optimize control measures. Prospective studies that consider pre-outbreak predictors, such as connecting networks, are recommended.

## Introduction

The first recorded effort of a successful intervention aimed at controlling an epidemic was that of John Snow –the British physician who, in 1854, discovered and prevented the dissemination mechanism of cholera epidemics [1]. Snow integrated what, today, could be described as medicine, statistics, geography, civil engineering, and cost-benefit analysis: he mapped London’s water network and, with simple graphs, quantified the number of cholera cases associated with specific households (http://en.wikipedia.org/wiki/File:Snow-cholera-map.jpg). That led him to geographically identify the water pump suspected to be contaminated. By removing the handle of the pump –leaving it non-operational–, he stopped the epidemic.

He did not intervene on people. He did not intervene on the pathogen. He intervened on a physical structure that connected susceptible hosts with the microbe –the water distribution network– and did so before infections could occur. Snow acted on connectivity, a concept related to, but independent from both the infectious agent and the susceptible host.

His example provides a reference against which views on how epidemics spread can be analyzed. Hoping that a review of fields involved in epidemiology may identify unmet research needs, the contents of mathematical epidemiology and medical geography are summarized.

*Mathematical epidemiology* focuses on hosts. It asks who is in contact with whom. [2], [3]. This field began in 1908, when Sir Ronald Ross, after discovering that mosquitoes transmit malaria, defined the ‘critical mosquito density’ (later known as the basic reproductive number, or R_0_ [4]. The R_0_ is the ratio of secondary cases generated per primary case which, if >1, indicates that the epidemic will disseminate; and, if ≤1, predicts that the epidemic will soon die out [5]. This approach has been applied both in endemic and in epidemic diseases [6], [7]. While, in some cases, this quantity or R_0_-related quantities are directly estimated from epidemic data [8], [9], R_0_ is usually indirectly estimated, utilizing a process whose validity depends on several assumptions [10]. One such assumption is that individuals are homogeneously mixed: the R_0_ concept may be valid when hosts are in close contact with one another. Yet, R_0_-based models, which do not consider low-scale geographical data, have overestimated some epidemics [11]–[18].

Other mathematical approaches have focused on *social structure* [2]. They consider sub-populations suspected to be the target of the epidemic, which could be under-estimated if the highest scale (the total population) is measured but no stratification is conducted [19]. Variations of this approach assess groups, e.g., family members, co-workers, and schoolmates [2]. These models do not consider geographical data.

A third group of mathematical models has explored *networks*. They do not assume that the population is homogeneously mixed. Instead, they consider the relative location of each individual (a ‘node’ or vertex, which may be represented by a circle or point), and contacts between individuals (‘links’ or ‘edges’, e.g., a line that connects two nodes [20]–[24]). While network models are usually labeled ‘spatial’, typically, they lack geographic data [25].

*Social network analysis* (SNA) is one exception to the previous statement. This approach may include geographically explicit data, as well as temporal data. It determines the location of individuals (‘nodes’) and the time and duration of contacts [26]. SNA has demonstrated that temporal structures may influence epidemics in several ways [27]. SNA has been reported to: 1) risk missing data on connections [27], and 2) be sensitive to dynamic changes [28].

*Medical geography* addresses some of the limitations described above. This approach is based on disease maps, today generated with geographical information systems. Such maps may reveal geographical data patterns likely to be missed when only tabular data are considered [29]. Geographical models are indicated when geographical heterogeneity is documented: when disease clusters (geographical aggregations of cases at higher levels than expected) are observed, homogeneous mixing-based models are not valid [15]. Coupled with spatial statistical analysis, disease maps have attempted not to explain general problems but to be applicable [30]. Potential limitations of this approach include: 1) dependence on a relatively large sample size (rarely available in the early phase of exotic epidemics), and 2) dependence on static processes (a rare event in emerging epidemics, in which, the centroid of disease clusters, may rapidly change).

To control epidemics, functional (network theory-based), geographically explicit models that measure both dynamics and connectivity are needed [31], [33]–[36]. Calls to study both global and local dynamics –which occur at high and low scales, respectively– have been expressed [15], [36]. Yet, the simple combination of the previous models will not generate what is needed because they focus on contacts (people or animals) and, at the earliest epidemic phase, the number of infected individuals is very low. While air-borne epidemics have been investigated [12], [37], they are atypical because their connecting structure is mobile, and, in air travel-mediated epidemics, reduced to the few yards that separate passengers sharing the same aircraft.

Therefore, a model that measures epidemic connectivity, is needed. Connectivity relates to, but differs from distance [38]–[40], for instance, two pairs of points, separated by the same Euclidean distance, will differ in connectivity if one pair is separated by a mountain or lake but the other pair is not. Connectivity can modify or be modified by distance, time, and/or neighbors: different geographical sites may behave as nodes at different times; e. g., a factory may act as a node on week days, losing that condition on weekends, when a park may become a node. It has been proposed that, because the network’s architecture influences the global microbial invasion and/or mobility, connectivity needs to be measured and, because connections change over time, geo-temporal data should be assessed [41], [42]. These propositions have been documented: road or river networks can promote or delay disease spread [32], [43]–[49].

While several authors have called for methods that integrate network analysis with geographical data [27], [32], [50], the lack of low-scale geo-referenced data has been mentioned as an impediment [51], [52]. A second reason to be considered is that nature does not offer bio-geo-temporal equivalents of ‘nodes’ and ‘edges’: they should be created and validated. To build such constructs, the model to be created should: 1) utilize low-scale geo-temporal data; 2) consider both short- and long-range connections as well as geo-temporal dynamics, i.e., the geo-temporal progression of the epidemic should be clearly determined; 3) evaluate reproducibility; and 4) facilitate comparisons against alternatives, which may include cost-benefit metrics [33], [53], [54].

In addition, the model should distinguish contact-related from connectivity-related networks, as John Snow did. While both networks are associated, they are not synonymous: while mobile people or animals use non-mobile connecting networks –such as road, water, railroad networks; as well as food networks (e.g., markets) and energy networks (e.g., gas stations)–, such networks are built before they are used by humans and animals. Hence, the properties of connecting networks can be investigated even without data on humans or animals.

However, the physical connecting network, per se, is not the concept of interest: measuring roads or railroads, alone, will not provide information usable to control epidemics. The network of interest is dynamic and much larger: it involves *bio-geo-temporal connecting interactions*.

Accordingly, two models were evaluated: 1) one focusing on connectivity (in addition to contacts), and 2) one focusing on contacts, in which connectivity was not explicitly assessed, but neighbors were considered. Both approaches were tested utilizing geo-temporal datasets of emerging or exotic infectious diseases that affect vertebrates. The validity of the connectivity model was evaluated by asking whether network properties were revealed (such as disease clustering, assortatitivy, synchronicity, directionality, and Pareto’s’ 20∶80′ data distribution [12], [20], [55], [56]). The reproducibility was determined by investigating infectious diseases that differed in pathogen, host species, vertebrate class, geography, and time, but shared the fact that all hosts were susceptible prior to microbial invasion. The cost-benefit impact was estimated by comparing, across models, the total number of cases, observed at the end of the study period. By counting the number of cases these models captured, we expected that the role of connectivity could be determined. It was postulated that, if network properties were detected in two episodes of disease dispersal that affected different classes of vertebrates (mammals and birds) and involved different pathogens, it could then be inferred that such properties are independent of infective agent, infected host species, vertebrate class, and spatial location. We hypothesized that, to rapidly disseminate, invading microbes require not only susceptible hosts but also a pre-established connecting structure (e.g., a river network). While many networks may exist, we focused on the one reported to be used most of the time: the road network [53]. Here we asked, first, whether actual processes of infectious disease dispersal display network properties, and, second, if so, whether a connecting network –that of roads– can influence disease spread.

## Materials and Methods

### Bio-geo-temporal Data (Primary Variables)

The foot-and-mouth (FMD) and avian influenza H5N1 (AI) epizoonotics here analyzed, affected cows and chickens, respectively. They have been reported before [43], [45]. Geographical data included: 1) point (epidemic cases), 2) line (roads), and 3) surface (population density) data. An *epidemic case* was defined as any farm where, based on laboratory tests, at least one animal was diagnosed as infected. *Epidemic day* reflected the relative time, within the epizoonotic, when a case was reported. The analyzed datasets differed: while the FMD dataset included data on the location and size of infected and non-infected farms, the AI dataset did not include data on non-infected farms. While the AI dataset included temporal data on daily basis for all observations, the FMD dataset had aggregate temporal data between epidemic days 7 and 60.

### Description of Constructs (Secondary Variables)

The *epidemic node* was defined as the smallest circle that included: 1) >50% of all *cases* reported per viral transmission cycle (TC), except TC I [45], and 2) a *highway intersection*. The reason why the smallest possible circle was measured is due to the finite dimensions of the Earth: the number of nodes is inversely related to their size (if the radius of the node were as large as that of this planet, there would be only one node and no links). The reason why data reported in TC I were not considered was that no disease dispersal has yet occurred at that time, i.e., in order to disseminate over space, a pathogen needs a time period equal to, or longer than one TC. We considered the TC of the FMD virus to be 3 days and that of the AI virus to be 2 days [43], [45]. Assuming that *epidemic nodes* were circular, their critical radius was determined by counting, at each TC, the number of cases located inside and outside circles of various radii [45]. While *epidemic nodes* always included *cases*, *cases* could also be found outside such *nodes*.

*Highway intersection areas* were circles of radius equal to that of *epidemic nodes*, centered on intersections. They shared all aspects of *epidemic nodes*, except *epidemic cases*.

*Road segments* (lines) were components of the road network. When located within *epidemic nodes*, they were assumed to estimate short-range node degrees.

An *infective link* was any segment of an Euclidean graph that connected pairs of *epidemic cases*. Depending on the location of such cases and/or the relative location of *epidemic nodes, infective links* estimated long-range connectivity. When *cases* were outside *epidemic nodes* and there was no *epidemic node* between *cases*, *infective links* did not involve *epidemic nodes*. However, when either *epidemic cases* were located within *epidemic nodes* or such nodes were located between pairs of *cases*, *infective links* crossed *epidemic nodes*: in such situations, the number of *infective links* crossing a node’s surface estimated long-range node degrees [22].

*Node rank* was the number of *infective link(s)* that intersected each *epidemic node*, where ‘rank 1’ identified the node crossed by the largest number of *infective links* and ‘rank n’ was the node crossed by the smallest number of such links. It was assumed that all *infective links* were available from day 1 onward [57]. Therefore, node degrees were assessed with indicators that estimated short- and long-range connectivity: *road segments* and *infective links*, respectively.

The *distance between road intersections* was generated with an additional graph. It connected all highway intersections, regardless of the presence or absence of epidemic cases.

*Neighbors* or *contacts* were later cases found within circles of radius equal to that of the *epidemic node*, centered on the location of earlier cases. They estimated the contact model.

The difference between the two models was connectivity: not measured in the contact model, measured in the connectivity model. While the contact model focused on a post-outbreak variable (contacts, e.g., neighbors), the connectivity model assessed a pre-outbreak variable (roads). While the contact model evaluated circles centered on infected sites, the connectivity model investigated circles centered on the road network. While roads could be captured by the contact model, such inclusion was not intentional: the contact model, per se, did not measure connectivity. While the connectivity model measured contacts, the contact model did not consider how infected and susceptible individuals could be linked outside the original cluster: the contact model inherently assumed that the invading agent could jump from one place to another without using a geographically continuous, observable path. While disease spread may be mediated by wind, air travel, or migratory birds [58], such patterns were not substantiated in these epizoonotics [43], [45].

Borrowing metrics from civil engineering, connectivity was described by length, continuity, and/or proximity [59]. *Proximity* was defined as the Euclidean distance between pairs of road intersections. *Length* referred to that of road segments. *Continuity* described the degree of fragmentation, if any, the road segments found in epidemic nodes could reveal. By superimposing the layers described above, additional digital and graphic data were created.

### Software

Connectivity estimates (e.g., infective links) were calculated with either a proprietary algorithm, *ArcView GIS 3.3*, *ArcGIS Desktop 9.0*, and/or *ArcGis 9.3* (ESRI, Redlands, CA, USA). Geographical data and spatial statistical tests were processed with *ArcGis 9.3*. The GIS command *buffer* was utilized to create circles of various radii which were then used to *select by location* the infected farms located inside and outside such circles. The GIS commands *intersect, clip,* and/or *merge* were used to group variables of various shapes (e.g., points and polygons).

Other statistical tests were performed with *Minitab 15* (Minitab Inc., State College, PA, USA).

## Results

### Non-random Patterns and Determination of Epidemic Nodes

#### Clustering

**Figure 1:**
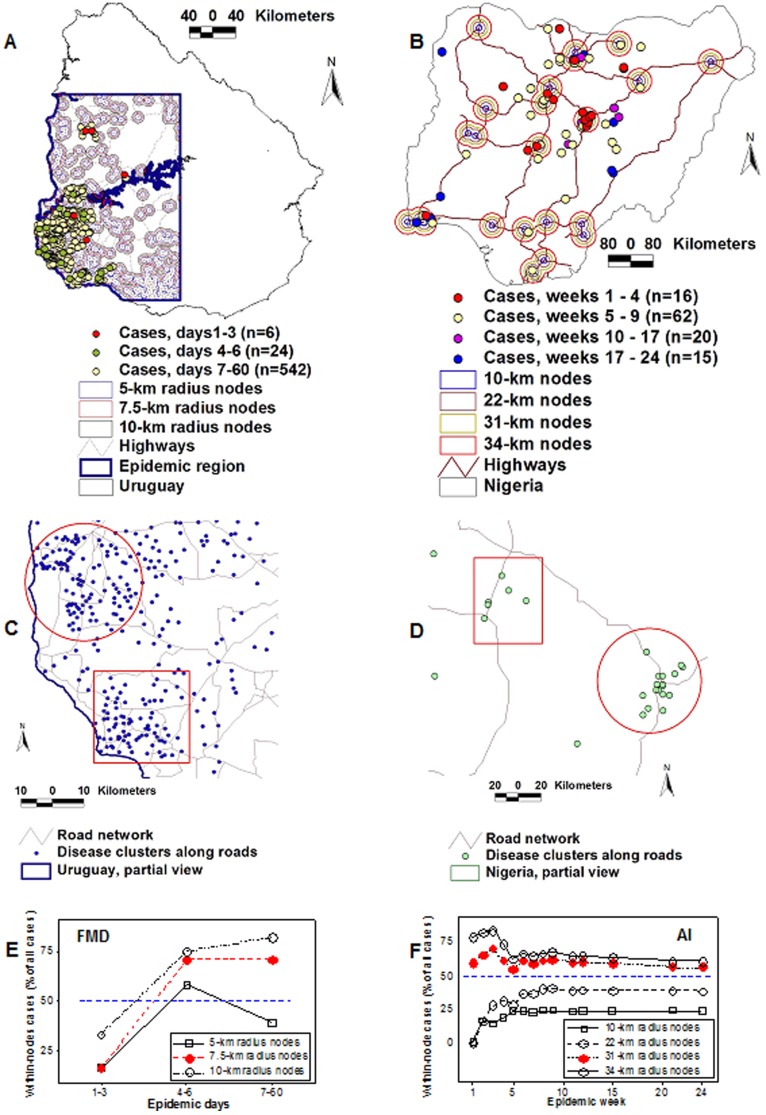
Detection of ‘along-road’ disease clusters and empirical determination of *epidemic nodes*. Maps show high-scale geographical data of the 2001 Uruguayan FMD (A) and the 2006 Nigerian AI H5N1 (B) epizoonotics. Low-scale data revealed that epidemic cases not only displayed spatial auto-correlation but also clustered along the road network (C, D).The radii of *epidemic nodes* (the smallest circles that included one or more highway intersections[s] and epidemic cases, at any viral transmission cycle [TC] except TC I) were 7.5 -km (FMD, E) and 31-km long (AI, F). In both epizoonotics, >57% of all cases occurred within epidemic nodes (A, B, E, F).

Both epizoonotics displayed spatial aggregation of *cases* (clustering). Although disease clusters are typically found only in early phases [60], they were detected over the whole disease dissemination process (60 days in FMD, 24 weeks in AI, Figures 1a, b). In addition to global and local case spatial auto-correlation [61] (*P*<0.01, Moran’s Index and Getis-Ord G, not shown), clustering was observed along roads, as expressed in Figures 1 c and d.

#### Validation of epidemic nodes

In the FMD epizoonotic, the smallest circle that included >50% of the cases, from the second transmission cycle (TC) onward, had a 7.5-km radius, while, in the AI epizoonotic, 31-km radius circles were the smallest that, at all times, included >50% of the cases (Figures 1e and f). Those circles, which included roads, estimated *epidemic nodes* (Table 1 in Text S1). *Epidemic nodes* included 57.5% (65/113, in AI) and 70% (402/572, in FMD) of all epidemic *cases*. These circles revealed epidemic dynamics: within 3 days (between TC I and TC II), the FMD epicenter (the centroid defined by all *epidemic nodes*) moved 40 km in a SW direction, while the centroid of the AI epizoonotic differed 700 km between the first and the second TC (Figure S1). Such nodes helped to reveal network properties.

### The FMD Network Properties and Discriminating Interactions

#### Differentiation of primary cases

**Figure 2:**
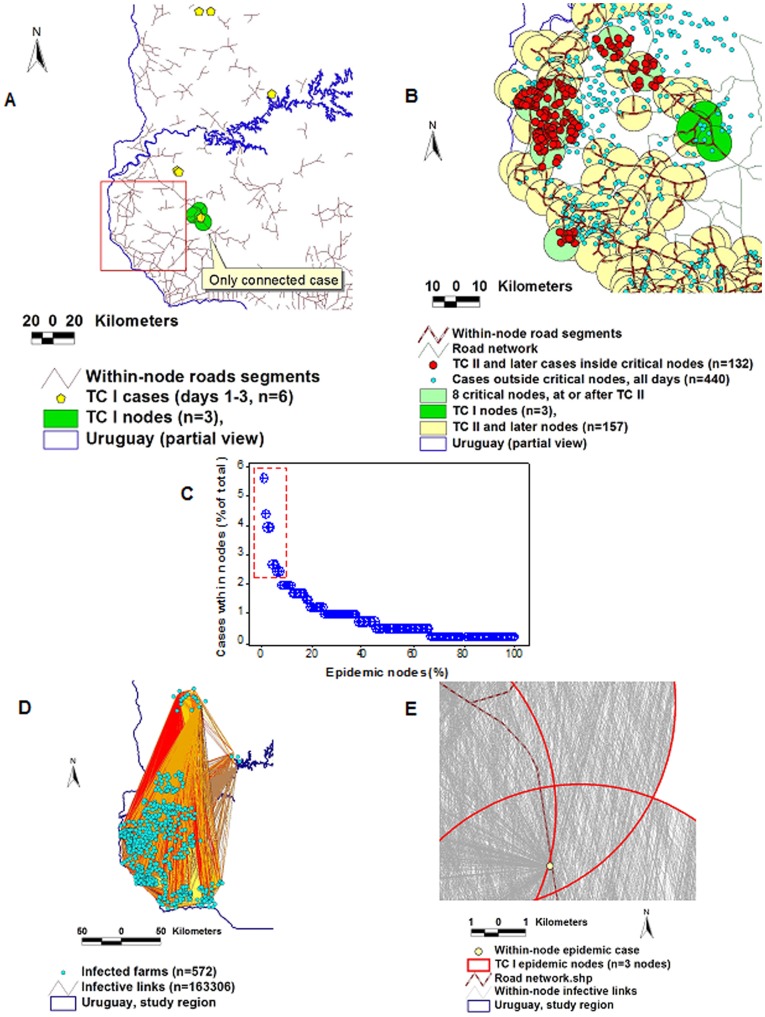
Differentiation of epidemic cases, detection of network properties, and estimation of long-range connectivity in the FMD epizoonotic. Not all primary FMD cases –those reported in the first transmission cycle or TC– were located within circles that included a highway intersection: only one the first 6 primary cases was connected (A). In contrast, at or after TC II, most cases were connected: they were within epidemic nodes. Some epidemic nodes included a much higher proportion of cases than average nodes, e.g., 8 epidemic nodes included 115 of all 402 within-node cases (B). Those 8 nodes were located in an area characterized by a high density of road segments (box, A). Such nodes revealed assortativity (selective connection among similar nodes) as well as Pareto’s “20∶80″ pattern: 8 of the 157 nodes connected at or after TC II (5% of all nodes) reported 23% of all cases (132/572), i. e., these nodes included 4.6 times (23/5) more cases than average nodes (B, C). To estimate long-range connectivity, a graph was made, which connected every pair of *epidemic cases* with Euclidean lines, here named *infective links* (D). A low-scale map shows *infective links* crossing 3 partially overlapping *epidemic nodes*, which include one *case* (E).

Only one of the 6 FMD *cases* (16.6%) reported in TC I (days 1–3) was found within *epidemic nodes* (Figure 2a). Therefore, not all primary *cases* were functionally identical: only one was connected. In contrast, in TC II (after the infectious agent had enough time to disseminate), 17 of the 24 cases (71%) were reported within *epidemic nodes* (Table S1). Hence, disease spread depended on getting access to a disseminating (connected) network, which was observable at or after TC II, as Figure 2b shows.

#### Differentiation of epidemic nodes and detection of Pareto’s pattern

*Epidemic nodes* were distinguished by the number of cases/node: 5% (8/157) of all *epidemic nodes* reported over 60 epidemic days included 23% of all *cases* (132/572, Table S1). That feature displayed a Pareto’s ‘20∶80 pattern’: a small percentage of nodes was associated with >4 times more cases (23/5 = 4.6) than expected under the assumption of an equal number of cases per node.

#### Assortativity

More connections were observed among similar than among dissimilar nodes (assortativity). Figures 2b and c indicate, both geographically and numerically, that, at or after TC II, 8 *epidemic nodes* displayed similarities: many *road segments* inter-connected such nodes, the 8 nodes were close to one another (some of them partially overlapped), and revealed a much higher percentage of *epidemic cases* than average nodes.

#### Synchronicity

The simultaneous engagement of functionally similar nodes was observed in TC II, when 56 FMD nodes were found to be connected (Figure S1).

#### Relationships between connectivity and case occurrence

**Figure 3:**
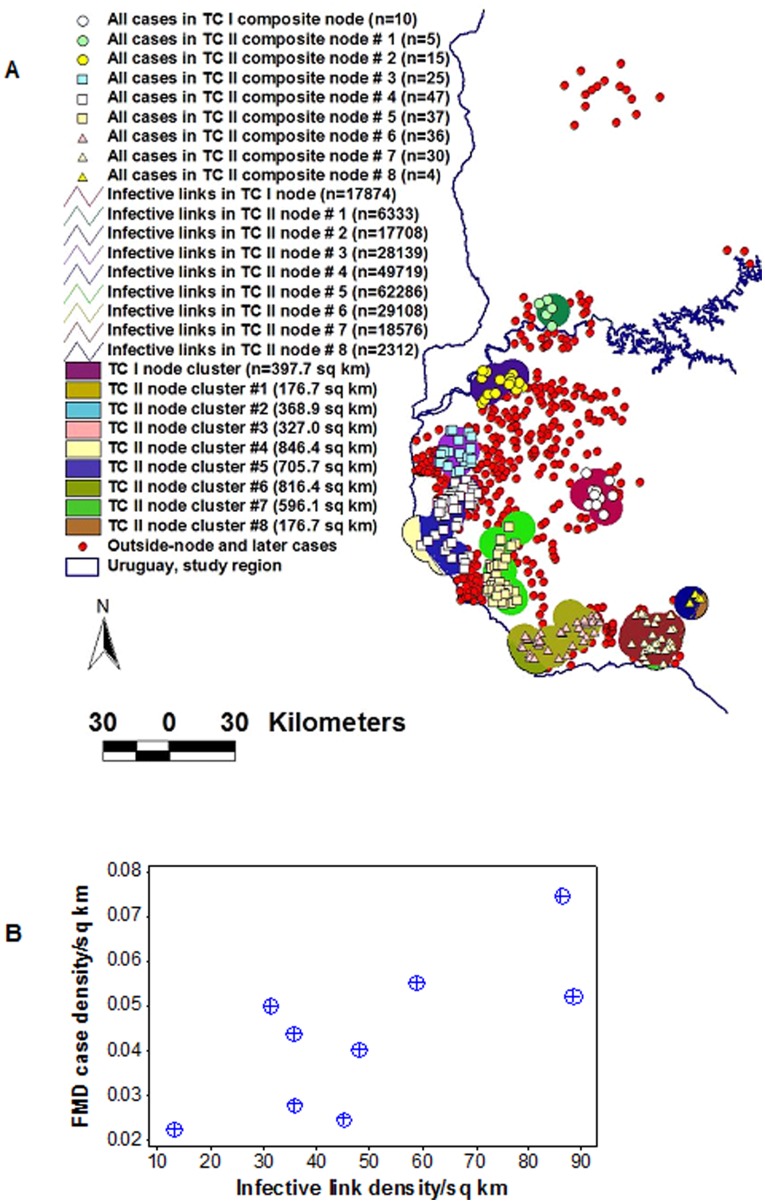
Relationships between pre- and post-outbreak variables in FMD. Because some TC I and TC II epidemic nodes overlapped, they were merged. Merging resulted in a total of 9 (one in TC I, 8 in TC II) node clusters (A). The hypothesis that the number of infective links crossing each node cluster preceded case occurrence was supported by the data: the correlation between *infective link density* (number of infective links crossing epidemic nodes, per sq km, observed at TC I and TC II) and within-node *case density* (cases reported by epidemic day 60, expressed on a per sq km basis) was positive and significant (*r* = .75, *P*<0.02, B). *Early* variables (*infective links* observed in the first 10% of the epidemic progression [days 1–6] predicted *late* outcomes (within-node case density, observed in the last 90% of the epidemic [days 7–60]).

Because some TC I and TC II *epidemic nodes* overlapped, such nodes were merged. Because FMD data, after TC II, were temporally aggregated, TC-specific node merging could not be conducted after TC II. To explore relationships between merged *nodes* (or *cases*) and *long-range connectivity*, a graph that connected every pair of epidemic *cases* was created. Figures 2 d and e express high- and low-scale versions of such graph, showing lines here named *infective links*. *Infective link density*/*node* (the number of TC I or II *infective links* crossing each [merged or non-merged] *epidemic node*, per sq km) was correlated with overall within-node *case density* (*r* = .75, *P<*0.02, Figures 3a and b, Table S2). That is, long-range connectivity, measured in the first 10% of the epidemic (days 1–6), predicted the density of cases/sq km found in the last 90% of the epidemic.

#### Relationships between connectivity and population density

**Figure 4:**
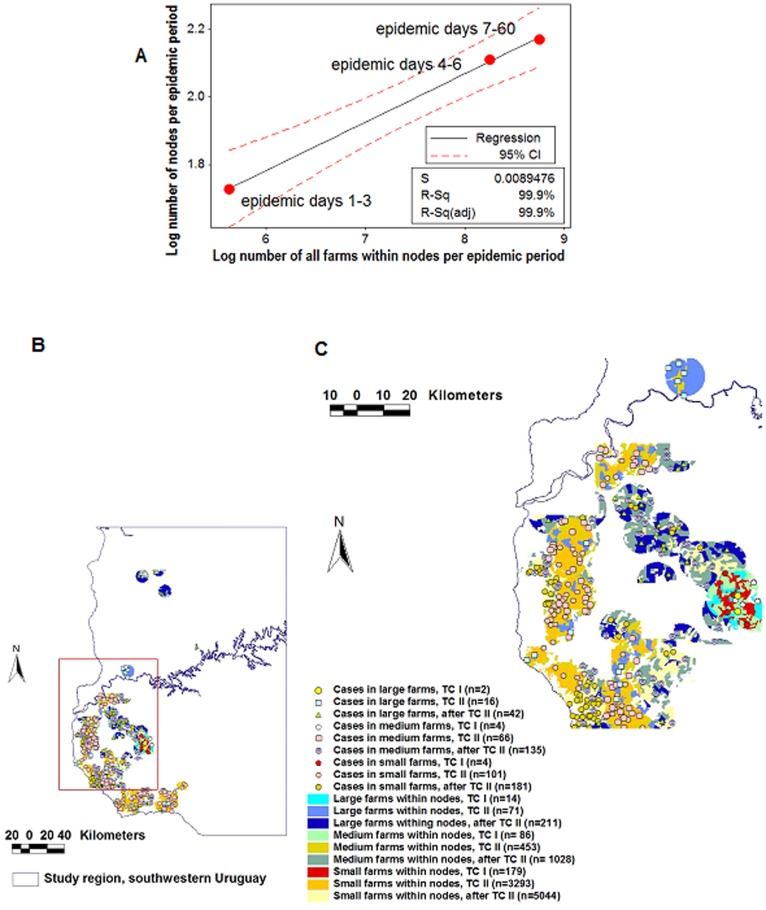
Dynamics of the FMD connectivity-population interaction. Farm density was used as a proxy variable for animal density. The *temporal connectivity* (*epidemic nodes* per *TC*) was positively correlated with the *temporal farm density* (characterized by size classes and measured per TC): over time, the greater the number of farms –which were smaller and raised more animals/sq km–, the greater the number of connected epidemic nodes found per TC (A). In spite of the observed correlation, a highly fractured (heterogenous) geographical distribution was observed (B, C). A subset of the whole epidemic region (indicated in a box shown in panel B) is displayed in panel C, which reports, numerically, the data of the region under study. Findings document that post-outbreak data (*cases*, *epidemic nodes*) can be linked to pre-outbreak (population, connectivity) data.

Farm density was assessed as a proxy estimate for animal density [62]. A global analysis showed that farm density was positively associated with the number of *epidemic nodes* per TC: over time, population density correlated with connectivity (Figures 4a–c). However, as Figure 4c reveals, that interaction was not a simple one but mediated by a heterogenous (fragmented) bio-geographical landscape.

### The AI Network Properties and Discriminating Interactions

#### Differentiation of primary cases and epidemic nodes, and detection of Pareto pattern

**Figure 5:**
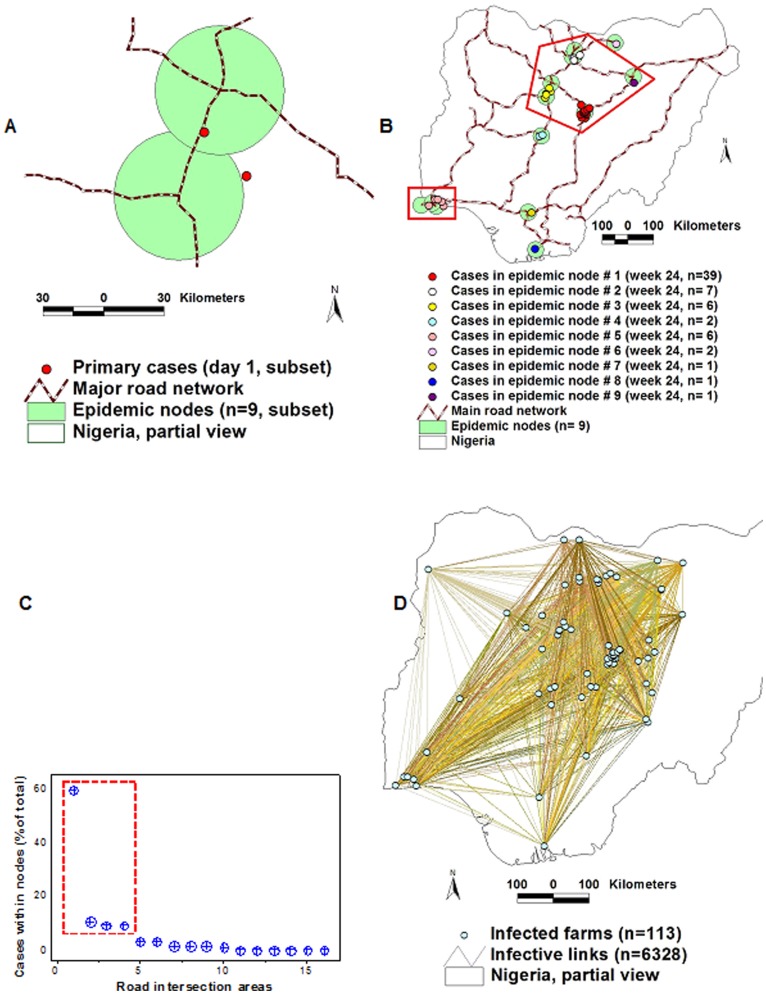
Differentiation of epidemic cases, detection of network properties, and estimation of long-range connectivity in the AI epizoonotic. Low-scale data revealed that one primary AI *case* was located close to but outside the connecting structure defined by *epidemic nodes* (A). In contrast, at or after TC II, most cases were found within epidemic nodes (B). Two *clusters* of *cases* were observed (red polygons, B). Some *epidemic nodes* displayed a much higher proportion of cases than average nodes, e.g., two nodes (nodes # 1 and 2, red pentagon, B) accounted for 46 (or 71%) of all within-node cases. Four *road intersection areas*, out of 16 (or 25%) included 80% (52/65) of all within-node *cases* (C). To estimate long-range connectivity, all pairs of epidemic cases were connected with Euclidean lines, conforming a graph of N * (N –1)/2 lines, where N = epidemic case (an infected farm), or (113 * 112)/2 = 6328 *infective links* (D).

Not all primary *epidemic cases* were connected. Figure 5a shows that not all primary cases were within *epidemic nodes*. *Epidemic nodes* were not functionally identical, either: four of them (44.4% of all nodes, red pentagon, Figure 5b) included 89% (58/65) of all within-node cases, i.e., 4 nodes showed a number of cases twice higher than average. Two of those nodes accounted for 46 within-node cases (red pentagon, Figure 5b). Hence, 22% (2/9) of all nodes explained 71% (46/65) of all within-node cases, i.e., a 3.3∶1 ratio –a Pareto pattern that also demonstrated not all *epidemic nodes* were similar. Epidemic node-associated clusters also met the criteria defined by Network Theory [63]: their road segments estimated short-range node degrees (Figure 5b).

#### Assortativity, interactions among networks, and a second Pareto pattern

Assortativity was visually observed: AI nodes # 1–3 showed the highest number of cases and linked with one another through a continuous ring of short-range road segments (red pentagon, Figure 5b). Interactions among networks were revealed: while 16 *highway intersections* were observed, only nine of them included *epidemic cases*, i.e., only 9 *road intersection areas* –a network composed of circles of radius equal to that of *epidemic nodes*– acted as *epidemic nodes* (Figure 5b). This network displayed a Pareto pattern: 25% of all *road intersection areas* (4/16) included 80% (52/65) of all within-node *epidemic cases* (Figure 5c).

#### Relationships between connectivity and case occurrence

**Figure 6:**
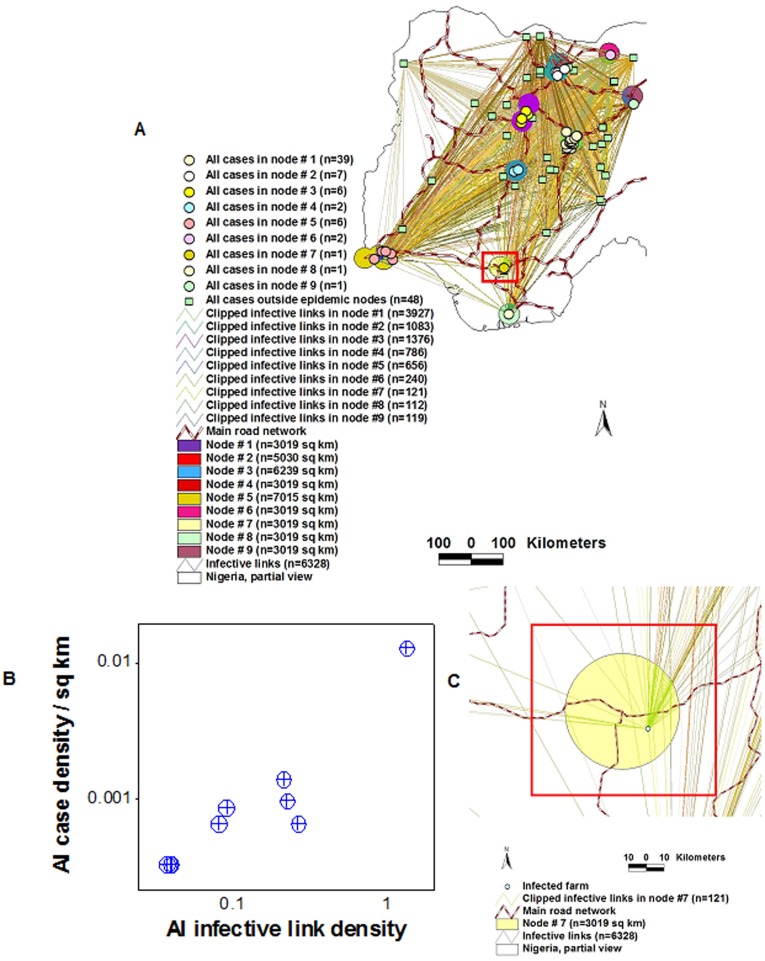
Differentiation of AI epidemic nodes based on AI infective links. After overlapping *epidemic nodes* were merged, they were distinguished according to the number of *infective links* that crossed their surfaces (A). The *density of infective links/node* was so high in nodes # 1–4 that the color used to identify each node’s circle is not observed: only the color of the crossing (overlaying) *infective links* is noticed in such nodes. The density of *infective links/epidemic node* (infective links/sq km) decayed by a factor greater than 5 between node #1 and the following set of nodes (nodes # 2 to 4), by a factor of ∼3 between nodes # 2–4 and the set that included nodes #5 and 6, and by a factor of ∼2 between nodes # 5 and 6 and the remaining nodes. A significant positive correlation was found between the *infective link density/sq km* and the *case density/sq km* (*r* = .98, *P*<0.001, B). An enlarged view of one AI epidemic node (red box, A), is shown in C.

*Infective link density/node* (links/sq km) correlated with *case density* (*r* = .98, *P*<0.001, Figures 6 a–c, and Table S2).

#### Synchronicity and directionality

**Figure 7:**
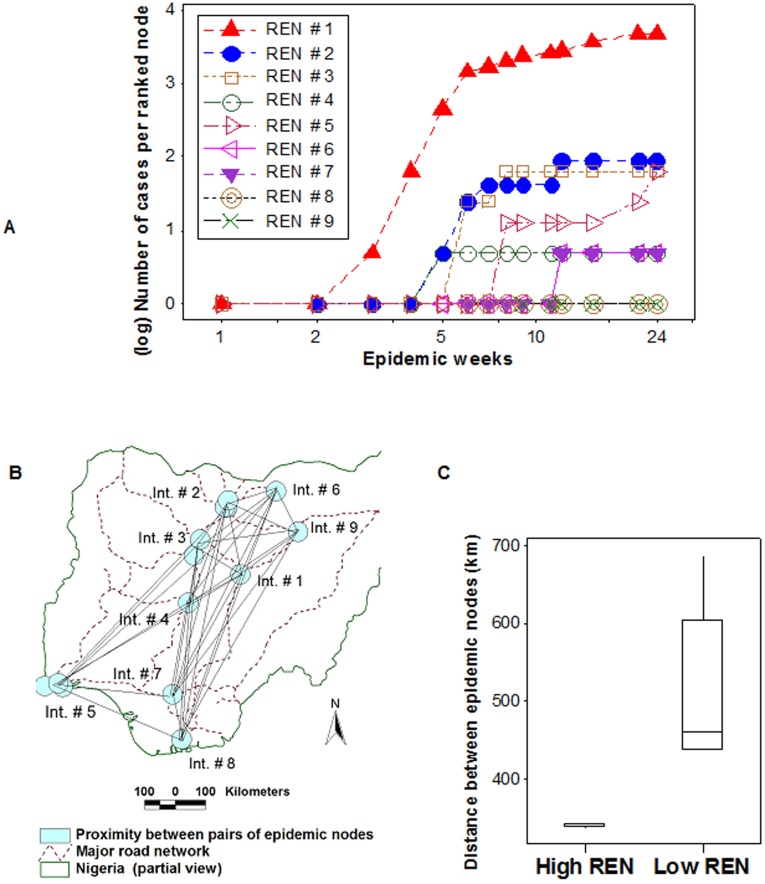
Synchronicity and directionality of AI epidemic flows and interactions between pre- and post-outbreak variables. Based on the data reported in Figure 6a, *epidemic nodes* were ranked according to the number of *infective links* that crossed their surface, e. g., *ranked epidemic node* (REN) # 1 was crossed by the highest number of infective links (**A**). Both synchronicity and directionality were revealed when RENs were plotted against the weekly (log) number of *epidemic cases*, and several classes of epidemic nodes were distinguished. REN # 1 was engaged first, and later, it was followed by nodes of lower ranks The epidemic flow moved from high to low RENs (directionality was observed) and, at a given point in time, similar nodes were active (synchronicity was demonstrated). RENs #8 and 9 had no influence on epidemic dispersal: they only produced one case each (**A**). An additional graph, which linked the centroids of *epidemic nodes*, determined the distance between pairs of highway intersection areas that included epidemic cases (**B**). The median distance between such intersections was significantly shorter for high than for low RENs (**C**). Such finding supported the view that critical hubs –connecting node structures, which predate epidemic occurrence and are likely to act as epidemic nodes– may be identified even before microbial invasions occur.

The number of *infective links*/*epidemic node* was used to rank nodes, e.g., *ranked epidemic node* (REN) # 1 was crossed by the highest number of *infective links*. When RENs were plotted against the number of *epidemic cases/*week, both synchronicity and directionality were observed. Figure 7a reveals that nodes of similar rank were engaged at the same time and high RENs were involved before low RENs. The number of *epidemic cases* grew rapidly in REN #1 and, when few or no new cases were reported, nodes of a lower rank (RENs # 2 and 3) became active, which displayed the same pattern and were followed, later, by nodes of an even lower functionality. In contrast, the last class of nodes failed to spread infections: RENs # 8 and 9 only generated one case each (Figure 7a).

#### Relationships between pre-outbreak and post-outbreak variables

The median *distance* between *epidemic nodes* (Figure 7b, and Table S3) was significantly shorter in high- than in low-rank nodes (Figure 7c). Hence, the shorter the distance between road intersections, the higher the chance that such intersections could spread disease, if an exotic microbe invaded.

### Cost-benefit Comparisons between Models

#### Performance in the FMD epidemic

**Figure 8:**
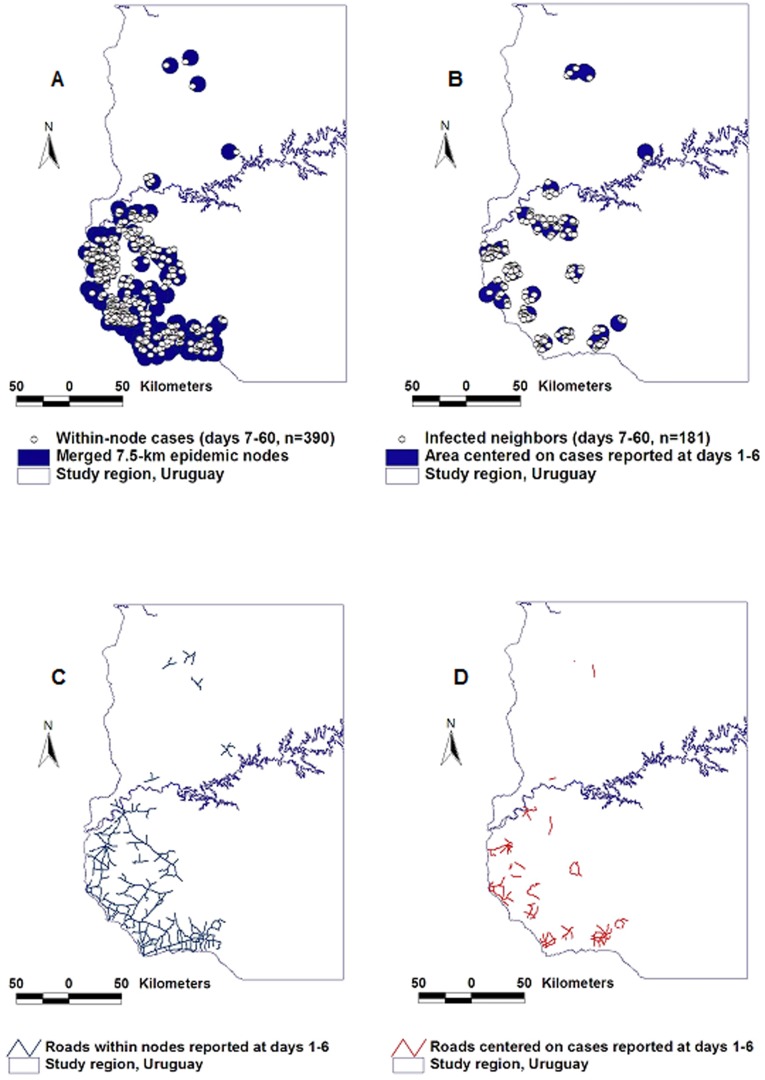
Comparison between connectivity and contact models–the FMD epizoonotic. After TC I, *epidemic nodes* centered on the road network (the *connectivity model*) showed twice as many cases as circles of equal radius that did not consider the road network (the *contact model*): while 360 cases were reported within epidemic nodes (A), 181 cases were found within the same time frame in the neighborhood of earlier cases (B). Longer *road length* and less fragmentated road segments were associated with the connectivity model (C) than with the contact model (D).

After TC II, 390 cases were included within *epidemic nodes* (Figure 8a). Within the same timeframe, 181 cases were reported within neighborhoods (circles of identical radius, centered on the location of all cases reported in the first two TCs, Figure 8b). FMD *epidemic nodes* were associated with a longer connectivity –longer road segments– and a more continuous structure than those of the contact model (Figures 8c, d).

#### Performance in the AI epidemic

**Figure 9:**
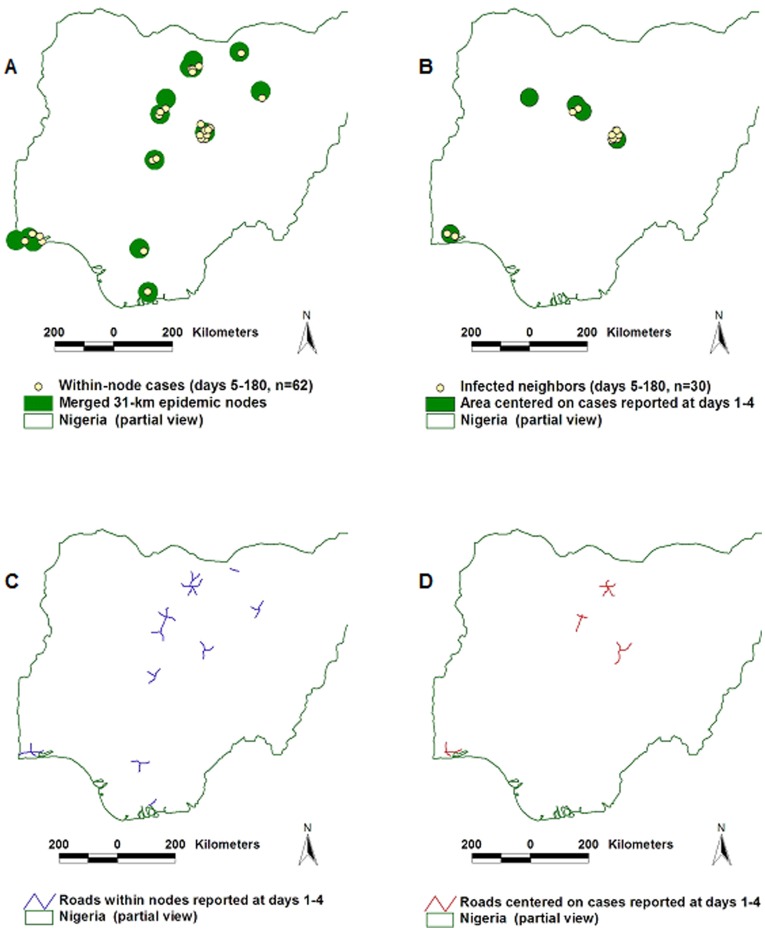
Comparison between connectivity and contact models–the AI epizoonotic. The AI dispersal process was similar to that of the FMD epidemic diffusion: after transmission cycle (TC) I, the connectivity model captured twice as many cases than the contact model (A, B). The length of road segments found within the area determined by the connectivity model was three times longer and less fragmented than the road structure captured by the contact model (C, D).

After TC II, more than twice as many *epidemic cases* (62/30) were found within the connectivity model than within circles centered on the location of earlier cases (Figures 9a, b). Figures 9c and d document that AI *epidemic nodes* had a 3-fold longer and less fragmented road structure than circles that did not consider connectivity.

#### Integration of spatial statistical, Network Theory, and bio-geo-temporal approaches

**Figure 10:**
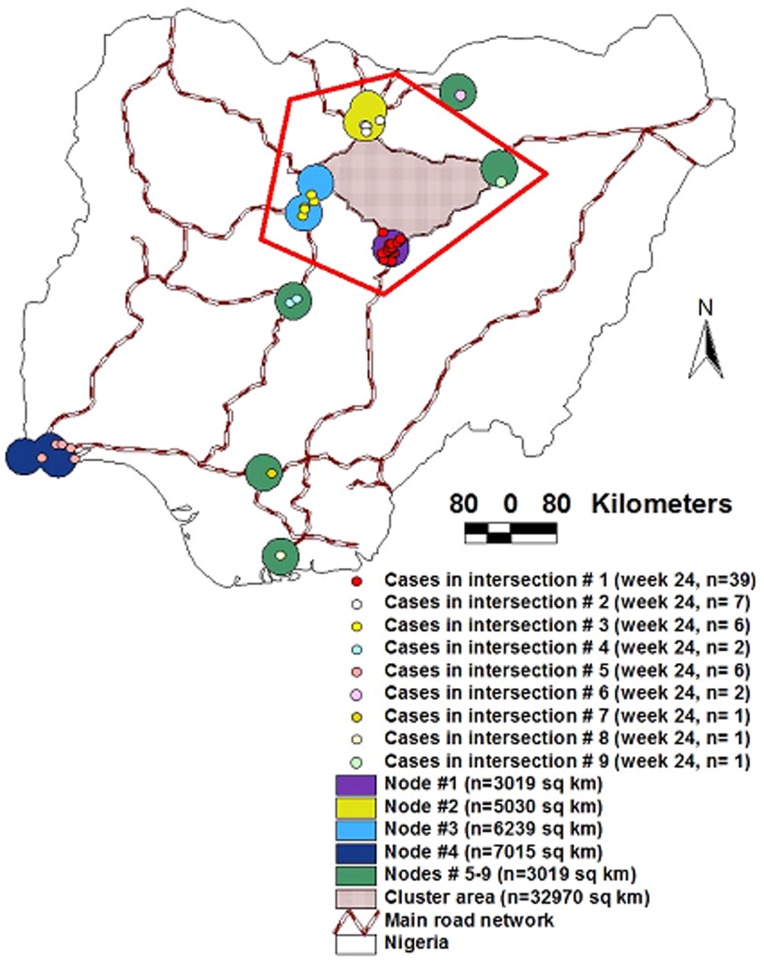
Three cost-benefit perspectives. The AI data allowed the generation of three sets of metrics, potentially applicable in cost-benefit analyses. 1) While the *spatial statistical* (SS) model identified *6 disease clusters* (the 6 *epidemic nodes*, of which two partially overlapped, which are seen, within the red pentagon, as 4 circles or ovals, of different colors), because the SS approach does not offer information on directionality, control measures should consider every *epidemic node*, i.e., the overall ‘cost’ of an intervention would be equal to the sum of the areas of the 6 original epidemic nodes included in the red pentagon. 2) If a *Network Theory* (NT) perspective were considered, only a *single cluster* would be observed (the area included within the red pentagon, which is defined by nodes and edges [road segments]). The NT model may generate several cost-benefit metrics. 3) A *bio-geo-temporal* analysis can integrate both SS advantages (a small area) and NT advantages (identification of the most influential node, based on analysis of network properties). The bio-geo-temporal model can generate the lowest ‘cost’ (smallest area to be intervened per each prevented case). Calculations are reported in the text.

The AI data allowed the generation of three sets of metrics, potentially applicable in cost-benefit analyses: 1) a *spatial statistical* (SS) approach, 2) a *Network Theory* (NT) version, and 3) a *bio-geo-temporal* alternative. While the SS version appeared to cover small circles (i.e., a low ‘cost’ per case), because it did not consider connectivity, control measures based on such approach should involve the cumulative areas of all such small circles. While the NT approach considered connectivity, it covered a much larger area than the SS model because, in NT, a cluster is defined in a different way: it includes both nodes and edges (links) which, together, define polygonal areas rather than small circles. These differences in concepts were visualized in Figure 10, which also showed that the bio-geo-temporal model integrated both SS and NT views, producing a better solution.

Under the SS model, *6 disease clusters* were found (*epidemic nodes* with, at least, two *cases* each, e.g., the 6 partially merged circles observed across Figure 10). In this model, the ‘cost’ of preventing a case, expressed as the area to be intervened, would be the sum of:

1. Cluster # 1 (3019 sq km/39 cases) = **77** sq km/case
2. Cluster # 2 (5030 sq km/7 cases) = **718** sq km/case
3. Cluster # 3 (6239 sq km/6 cases) = **1039** sq km/case
4. Cluster # 4 (3019 sq km/2 cases) = **1509** sq km/case
5. Cluster # 5 (7015 sq km/6 cases) = **1169** sq km/case
6. Cluster # 6 (3019 sq km/2 cases) = **1509** sq km/case

If, instead, *Network Theory* (NT) was considered, only a *single cluster* would be observed, which would be composed of *4 nodes* (the 4 partially merged *epidemic nodes* observed within the red pentagon, Figure 10). In this model, connectivity among nodes could be determined by inside- and outside-node road segments. At least three calculations could then be generated, e.g.: 1) if it was assumed that all within-node cases, of all nodes, would be protected if the *whole area of the cluster* was intervened, the ‘cost’/case would be  = 32970/65 = **507** sq km; 2) if it was assumed that optimal control depends on interventions covering *the area defined by epidemic nodes*, the ‘cost’ would be equal to the surface of nodes #1–4 (17,307 sq km)/number of cases within such nodes (54) or **320.5** sq km/case; or 3) if it was assumed that such intervention would prevent all within-node cases (including those outside the node cluster), then the ‘cost’ would be: the surface of nodes #1–4 (17,307 sq km)/all within-node cases (65) = **266.3** sq km/case.

A *bio-geo-temporal* analysis could integrate both SS advantages (a small area upon which interventions are imposed) and NT advantages (those associated with the application of NT properties, especially, identification of highly influential nodes and directionality). Such model could focus on the most influential node (ranked epidemic node [REN] #1), which had a surface equal to 3019 sq km). If NT holds, an early intervention on such node could prevent all within-node cases (n = 65) at a ‘cost’ of 3019/65 = **46** sq km/case (Figure 10).

## Discussion

Both epizoonotics revealed highly organized data structures [64]. In spite of differences in microbial agent, host species, vertebrate class, time, and geographical location, five network properties –disease clustering, assortative mixing, synchronicity, directionality, and Pareto’s pattern– were observed, which seemed to be highly conserved. Disease dispersal was better explained by the connectivity-based model: after TC II, this model captured twice as many cases as, and displayed a less fragmented and longer length of road segments than the contact model. Connectivity also distinguished functional classes of primary cases, nodes, and networks.

The enhanced discrimination achieved by the connectivity model was attributable to the use of two indicators: *epidemic nodes* and *infective links*. With these constructs, what previously seemed to lack ‘order’, became interpretable and revealed a major property of biological systems: *emergence* (‘order’ or a high-level function [65]). For instance, *emergence* was observed when the weekly number of *epidemic cases* was plotted vs. the rank of *epidemic nodes*. The plot shown in Figure 7a documents that the time, number, and place of case occurrence were not random events but the result of *epidemic nodes* differentiated by *infective links*.

The indicators evaluated only partially related to definitions utilized in spatial statistics (SS) and Network Theory (NT). There were differences between or among: 1) ‘spatial’ and ‘geographic’, 2) ‘mobility’ and ‘connectivity’, 3) ‘nodes’ (as defined in NT) and *epidemic nodes*, 4) ‘links’ (node degrees, as defined in NT) and *infective links*, 5) ‘clusters’ (as defined in SS and NT) and *disease clusters*, and 6) classes of *primary cases*, *networks,* and *epidemic nodes*.

The connectivity model revealed that not all *primary cases* were connected. That finding may explain why, in the past, R_0_ has overestimated some epidemics [16]: the inclusion of non-connected cases overestimates the number of primary cases. Because, in emerging infections (when all hosts are susceptible), secondary cases can only be generated by some of the primary case(s), tertiary cases can only be produced by primary or secondary cases and so on [66], it follows that epidemic cases are neither independent nor functionally identical: connected *primary cases* are much more influential than any later case. Instead of interventions based on identical control zones, centered on the location of all earlier cases –i.e., the contact model, which assumes that all earlier cases have an identical probability of disseminating the infection to their neighbors–, interventions could consider connected primary cases.

The data also differentiated several networks, which may overlay and interact with one another [67]. While related, they were not identical: the *epidemic node* network did not include all road intersections, and the *road intersection area* network did not include all cases.

The highly connected *disease clusters* found within *epidemic nodes* differed from both spatial statistical (SS) and Network Theory (NT) definitions. In SS, a *cluster* denotes a spatial aggregation of *epidemic cases* (point data, in this study), of unknown connectivity, which may or may not be located within *epidemic nodes*. In NT, a cluster refers to groups of *epidemic nodes* (circles, in this study) connected by *road segments*. In other words, the NT version of a cluster, which is based on the clustering coefficient [63], is geographically larger than the SS version. On the other hand, the SS version of a cluster cannot identify critical nodes.

These different definitions and limitations were visually observed. For instance, Figure 10 displays either a *single cluster* composed of 4 epidemic nodes –the NT version of a cluster (red pentagon)–, or, in the SS version, *6 clusters* (the 6 epidemic nodes that include, each, two or more cases). While compatible with both the SS and NT approaches, the bio-geo-temporal model was more informative: if two disease clusters displayed identical SS indices (e.g., Moran’s I) or identical NT cluster coefficients [61], the bio-geo-temporal approach could distinguish them in terms of *continuity*, *long-range connectivity*, *proximity*, and/or *transmission cycle*.

Because classic NT models only consider tabular and continuous data, critical geographical features –which may be fragmented or discontinuous– may be missed. Such features can be measured by the *bio-geo-temporal* approach which, it addition, can estimate both directionality (not measured by SS models) and low- and high-scale geographical variables (not measured by NT models). Because the bio-geo-temporal model also revealed disease clusters with outbound flows, earlier views on disease clusters, which assume disease clusters are only recipients of infective flows [68], could be revised.

Differentiating the functional role of epidemic nodes is crucial to identify not only where, but also when an intervention can be most successful. Defining the ‘critical response time’ (time available to implement an intervention and achieve the results such intervention promotes [9]) is meaningful only if associated with information on where control measures can be applied.

Such geo-temporal information was provided in this study because *epidemic nodes* were distinguished. Node differentiation was possible because connectivity was not regarded to be synonymous with mobility. While the non-geographical literature assumes that mobility (the movement of people or animals, i.e., ‘contacts’) is equal to connectivity, that literature does not assess the structure that facilitates mobility. While ‘contacts’ are mobile, the connecting structure (e.g., the riverbed of a river network) is not. This distinction has practical effects: because in early disease dissemination phases, the number of infected ‘contacts’ (mobile individuals) is close to zero, the ‘contact’ version of connectivity (mobility) cannot be applied at such time. However, because there is no shortage of data on the connecting network (e. g., a road network), early and geographically contextualized calculations can be implemented when the focus of the analysis –and that of interventions– is the non-mobile connecting network, as John Snow did.

The fact that network properties may be observed in rapidly disseminating infectious diseases, in which the number of early cases is marginal –when information is most needed–means that, instead of focusing on the host (e.g., case counts), better decisions could be made if based on connectivity. To that end, Network Theory concepts were adjusted to bio-geo-temporal formats. To facilitate cost-benefit analyses, the definitions of *epidemic node* and *disease cluster* differed from those of Network Theory (NT). While nodes, in NT, are defined as dimensionless points [69], *epidemic nodes* were defined as surfaces (the smallest circle containing most cases). While, in NT, ‘cluster’ refers to sets of ‘nodes’, here overlapping nodes were merged, i.e., a *disease cluster* involved both *cases* and *nodes*. Such adjustments of NT concepts to bio-geographical realities generated both the lowest cost (interventions applied to the smallest circle) and the highest benefit (more epidemic cases could be prevented), as figure 10 shows. Because pre-outbreak data significantly correlated with post-outbreak findings, such as the positive and significant relationship found between *infective link density/node* and *case density*, if geo-referenced data on all susceptible sites –farms, in this study– were available, bio-geographical variables could, potentially, be measured before a microbial invasion occurs.

While the validity of *epidemic nodes* and *infective links* was supported, their limitations should not be ignored. *Infective links* assumed that connectivity remains constant over the course of an epidemic, which is unlikely [27]. While *epidemic nodes* detected ‘along-road’ disease clusters even if they were not independent –an advantage over classic approaches [70]–, such nodes, here assumed to be circular, may not be realistic. To improve such constructs, future studies may consider non-circular and non-Euclidean metrics, such as *road segments* and the *road length* associated with each node –here measured but only partially evaluated.

While disease spread may be mediated by other means, rapidly disseminating epizoonotics appear to require pre-established connecting networks. The integration of John Snow’s approach –interventions neither applied on the host nor imposed on the pathogen, but centered on connectivity– with network analysis, seems to be feasible.

## Supporting Information

Figure S1 **Number and location of epidemic nodes and centroid of epidemic nodes per epidemic days (DOC).**
(DOC)

Table S1 **Determination of epidemic node radius and number of epidemic cases over time (DOC).**
(DOC)

Table S2 **Relationships between infective link density and case density (DOC).**
(DOC)

Table S3 **AI H5N1 infective links, ranked epidemic nodes, and Euclidean distance between pairs of ranked road intersection areas (DOC).**
(DOC)
