# Supplementary material for: Connecting Network Properties of Rapidly Disseminating Epizoonotics
Source: PLoS One. 2012 Jun 25;7(6):e39778. doi: 10.1371/journal.pone.0039778 (PMC3382573; doi:10.1371/journal.pone.0039778)
Supplement: Figure S1 — Number and location of epidemic nodes and centroid of epidemic nodes per epidemic days (DOC). (DOC) [file pone.0039778.s001.doc]

**Figure S1.** **Number and location of epidemic nodes and centroid of epidemic nodes per epidemic days**. **A**: FMD. **B**: AI H5N1.


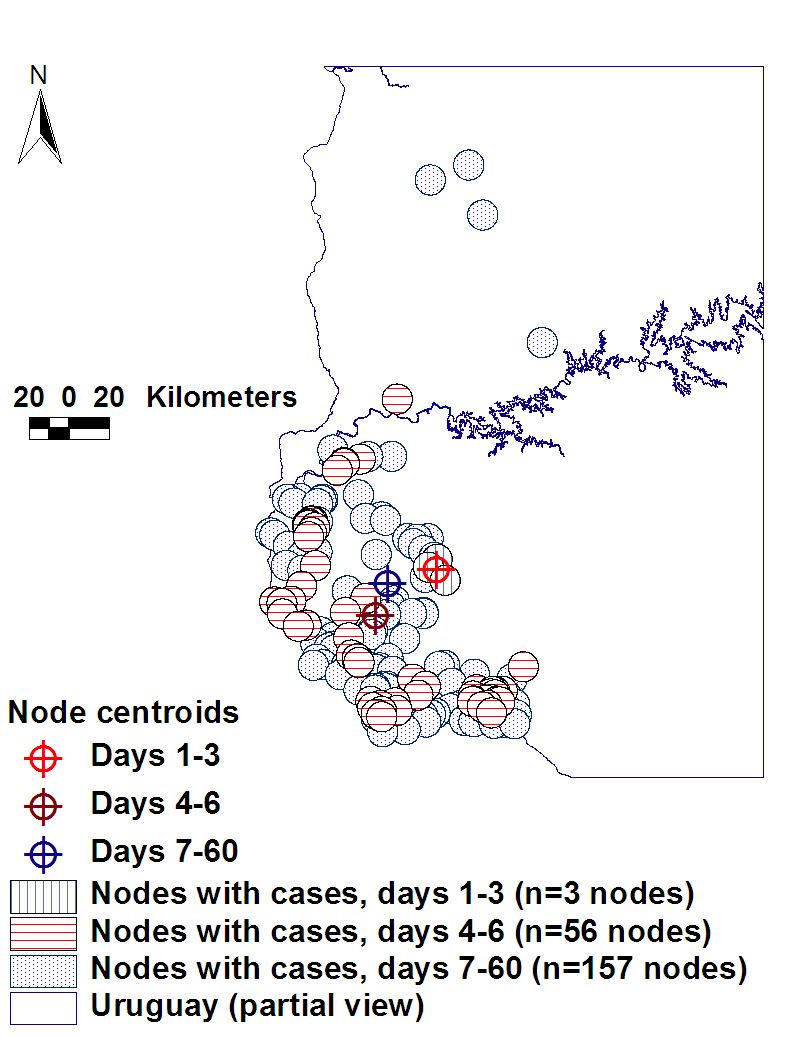


**A**


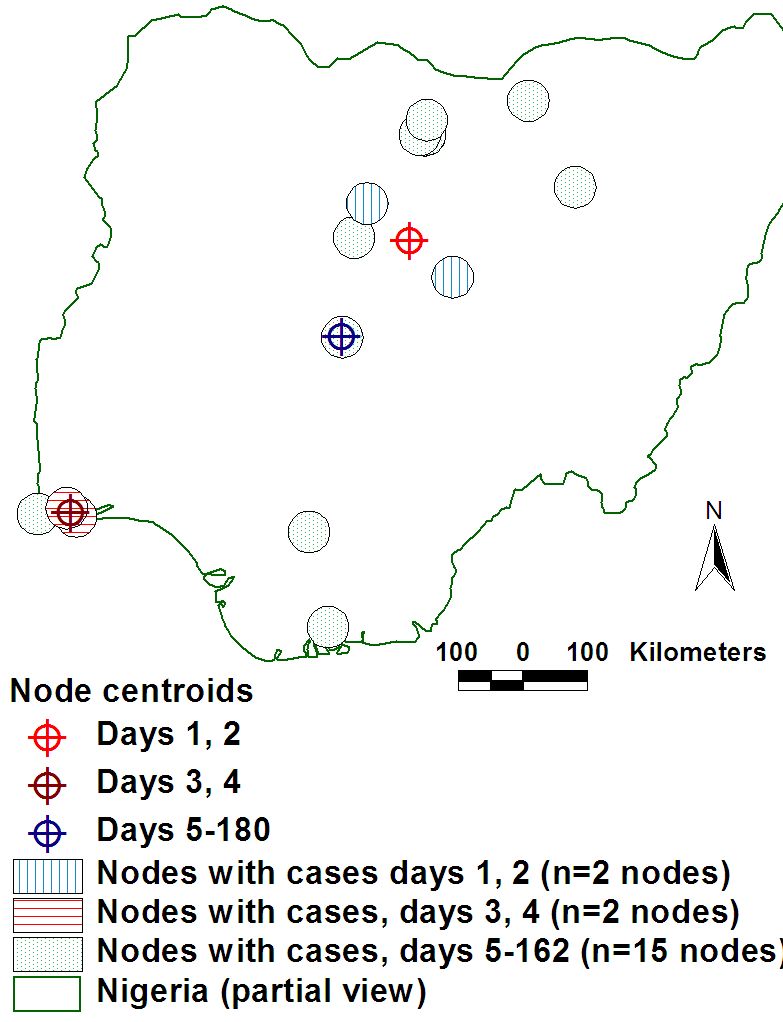


**B**
