# Supplementary material for: Connecting Network Properties of Rapidly Disseminating Epizoonotics
Source: PLoS One. 2012 Jun 25;7(6):e39778. doi: 10.1371/journal.pone.0039778 (PMC3382573; doi:10.1371/journal.pone.0039778)
Supplement: Table S1 — Determination of epidemic node radius and number of epidemic cases over time (DOC). (DOC) [file pone.0039778.s002.doc]

**Table S1. Determination of epidemic node radius and number of epidemic cases over time**

| **FMD** | n=572 cases |  | | | | | | | | | |
| --- | --- | --- | --- | --- | --- | --- | --- | --- | --- | --- | --- |
| Epidemic day | Cases inside  5-km  nodes | Cases outside  5-km nodes | | Cases inside 7.5-km nodes | | Cases outside 7.5-km nodes | Cases inside  10-km nodes | Cases outside 10- km nodes | Case % within 5-km nodes | Case % within 7.5-km nodes | Case % within 10-km nodes |
| 1-3 | 1 | 5 | | 1 | | 5 | 2 | 4 | 16.7 | 16.7 | 33.3 |
| 4-6 | 14 | 10 | | 17 | | 7 | 18 | 6 | 58.3 | 70.8 | 75 |
| 7-60 | 212 | 330 | | 384 | | 158 | 446 | 96 | 39.1 | 70.8 | 82.3 |
| Ratio TC II/TC I |  | | | | | | | | 3.49 | 2.44 | 2.25 |
| **AI H5N1** | n=113 cases | |  | | | | | | | | |
| Epidemic week | Cases inside 22-km nodes | | Cases outside 22-km nodes | | Cases inside 31-km nodes | Cases outside 31-km nodes | Cases inside 34-km nodes | Cases outside 34-km nodes | Case % within 22-km n. | Case % within 31-km n. | Case % within 34-km n. |
| 1 | 0 | | 5 | | 3 | 2 | 4 | 1 | 0 | 60 | 80 |
| 2 | 1 | | 5 | | 4 | 2 | 5 | 1 | 16.6 | 66 | 83.3 |
| 3 | 2 | | 5 | | 5 | 2 | 6 | 1 | 28.5 | 71.4 | 85.7 |
| 4 | 5 | | 11 | | 10 | 6 | 12 | 4 | 31.2 | 62.5 | 75.0 |
| 5 | 11 | | 27 | | 21 | 17 | 24 | 14 | 28.9 | 55.3 | 63.1 |
| 6 | 22 | | 37 | | 37 | 22 | 40 | 19 | 37.2 | 62.7 | 67.8 |
| 7 | 25 | | 42 | | 40 | 27 | 44 | 23 | 37.3 | 59.7 | 65.6 |
| 8 | 30 | | 44 | | 46 | 28 | 50 | 24 | 59.4 | 62.1 | 67.5 |
| 9 | 32 | | 46 | | 49 | 29 | 53 | 25 | 41.0 | 62.8 | 67.9 |
| 11 | 32 | | 50 | | 50 | 32 | 54 | 28 | 64.0 | 60.9 | 65.8 |
| 12 | 35 | | 53 | | 54 | 34 | 58 | 30 | 39.7 | 61.3 | 65.9 |
| 15 | 38 | | 58 | | 58 | 38 | 62 | 34 | 39.5 | 60.4 | 64.5 |
| 21 | 43 | | 67 | | 63 | 47 | 68 | 42 | 39.0 | 57.2 | 61.8 |
| 24 | 44 | | 69 | | 65 | 48 | 70 | 43 | 38.9 | 57.5 | 61.9 |
| Ratio TC II/TC I |  | | - | |  |  |  |  | No data in TC I | 1.1 | 1.0 |
