# Supplementary material for: Connecting Network Properties of Rapidly Disseminating Epizoonotics
Source: PLoS One. 2012 Jun 25;7(6):e39778. doi: 10.1371/journal.pone.0039778 (PMC3382573; doi:10.1371/journal.pone.0039778)
Supplement: Table S2 — Relationships between infective link density and case density (DOC). (DOC) [file pone.0039778.s003.doc]

**Table S2. Relationships between infective link density and case density**

| FMD | Early variables* | | | Late variables* | | |
| --- | --- | --- | --- | --- | --- | --- |
|  | Composite node area (sq km) | Infective links per composite node | Infective link density/node  (links/sq km) | Cases in composite node at the end of the epidemic | Case density (cases/ sq km) | |
| TC I node | 397.7 | 17874 | 44.94 | 10 | .0251 | |
| TC II node #1 | 176.7 | 6333 | 35.84 | 5 | .0282 | |
| TC II node #2 | 368.9 | 17708 | 48.00 | 15 | .0406 | |
| TC II node #3 | 327.0 | 28139 | 86.05 | 25 | .0746 | |
| TC II node #4 | 846.4 | 49719 | 58.74 | 47 | .0555 | |
| TC II node #5 | 705.7 | 62286 | 88.26 | 37 | .0524 | |
| TC II node #6 | 816.4 | 29108 | 35.65 | 36 | .0440 | |
| TC II node #7 | 596.1 | 18576 | 31.16 | 30 | .0503 | |
| TC II node #8 | 176.7 | 2312 | 13.08 | 4 | .0226 | |
| Correlation FMD infective link density vs. FMD case density *r* = 0.75, *P <* 0.02 | | | | | | |
| AI | Early variables* | | | Late variables* | | |
|  | Composite node area (sq km) | Infective links per composite node | Infective link density/node  (links/sq km) | Cases in composite node at the end of the epidemic | | Case density (cases/ sq km) |
| Node # 1 | 3019 | 3927 | 1.30 | 39 | | .01291 |
| Node # 2 | 5030 | 1083 | 0.21 | 7 | | .00139 |
| Node # 3 | 6239 | 1376 | 0.22 | 6 | | .00096 |
| Node # 4 | 3019 | 786 | 0.26 | 2 | | .00066 |
| Node # 5 | 7015 | 656 | 0.09 | 6 | | .00085 |
| Node # 6 | 3019 | 240 | 0.08 | 2 | | .00066 |
| Node # 7 | 3019 | 121 | 0.04 | 1 | | .00033 |
| Node # 8 | 3019 | 112 | 0.037 | 1 | | .00033 |
| Node # 9 | 3019 | 119 | 0.039 | 1 | | .00033 |
| Correlation AI infective link density vs. AI case density *r* = 0.986, *P <* 0.001 | | | | | | |

* Due to merging of partially overlapping *epidemic nodes*, the area of composite (merged) epidemic nodes differed across such nodes. The number of *infective links* crossing each node refers to *nodes* showing epidemic *cases* at the transmission cycle (TC) indicated (FMD) or at any time throughout the epidemic (AI). The reason for such difference in criteria is that, in the FMD epidemic, only aggregate data were available after TC II: because epidemic nodes activated between days 7 and 60 overlapped and no temporal data were available, it could not be distinguished when such nodes became activated. Hence, the comparison between infective links and case densities, in the FMD epidemic, only involves epidemic nodes reported in the first two TCs. In both epidemics, *case density* refers to the total number of within-node cases, observed at the end of the epidemic, which was expressed in relation to the area of such nodes.
