# Supplementary material for: Connecting Network Properties of Rapidly Disseminating Epizoonotics
Source: PLoS One. 2012 Jun 25;7(6):e39778. doi: 10.1371/journal.pone.0039778 (PMC3382573; doi:10.1371/journal.pone.0039778)
Supplement: Table S3 — AI H5N1 infective links, ranked epidemic nodes, and Euclidean distance between pairs of ranked road intersection areas (DOC). (DOC) [file pone.0039778.s004.doc]

**Table S3. AI H5N1 infective links, ranked epidemic nodes, and Euclidean distance between pairs of ranked road intersection area**s

| REN # (number of infective links)* | REN 1  (3927) | REN 2  (1083) | REN 3  (1376) | REN 4 (786) | REN 5  (656) | REN 6  (240) | REN 7  (121) | REN 8  (112) | REN 9  (119) |
| --- | --- | --- | --- | --- | --- | --- | --- | --- | --- |
| Distance between RENs (km)* |  |  |  |  |  |  |  |  |  |
| distREN 1 | -- |  |  |  |  |  |  |  |  |
| distREN 2 | 240.2 |
| distREN 3 | 184.1 | 170.8 |
| distREN 4 | 203.5 | 343.2 | 192.5 |
| distREN 5 | 717.1 | 806.9 | 687.9 | 517.3 |
| distREN 6 | 342.8 | 166.6 | 324.2 | 472.1 | 975.8 |
| distREN 7 | 452.3 | 646.7 | 504.5 | 318.2 | 393.5 | 751.6 |
| distREN 8 | 576.7 | 776.2 | 616.9 | 451.1 | 466.8 | 879.9 | 158.5 |
| distREN 9 | 241.1 | 248.6 | 337.2 | 438.6 | 951.8 | 172.2 | 671.4 | 795.6 | -- |
| Median | 342.8 | 343.2 | 337.2 | 343.2 | 687.9 | 472.1 | 452.3 | 576.7 | 438.6 |

* This table shows the distance between pairs of ranked road intersection areas (distRENs). Ranked epidemic nodes (RENs) are identified based on the decreasing number of infective links/ epidemic node, e.g., REN #1 (left column) was crossed by the highest number of infective links (shown on the top row) and REN# 10 was crossed by the lowest number of infective links. The graphic version of this table is shown in Figure 7b.
